# Synthesis, Characterization and the Solvent Effects on Interfacial Phenomena of Jatropha Curcas Oil Based Non-Isocyanate Polyurethane

**DOI:** 10.3390/polym9050162

**Published:** 2017-05-01

**Authors:** Mhd. Abd. Cader M. Haniffa, Yern Chee Ching, Cheng Hock Chuah, Yong Ching Kuan, De-Shin Liu, Nai-Shang Liou

**Affiliations:** 1 Department of Chemical Engineering, Faculty of Engineering, University of Malaya, 50603 Kuala Lumpur, Malaya; cader@siswa.um.edu.my; 2 Department of Chemistry, Faculty of Science, University of Malaya, 50603 Kuala Lumpur, Malaya; chchuah@um.edu.my; 3 University of Reading Malaysia, Persiaran Graduan, Kota Ilmu, Educity, 79200 Iskandar Puteri, Johor, Malaysia; K.Y.Ching@reading.edu.my; 4 Department of Mechanical Engineering, National Chung-Cheng University, Chia-Yi 62102, Taiwan; liu@ccu.edu.tw; 5 Department of Mechanical Engineering, Southern Taiwan University of Science and Technology, Tainan City 710, Taiwan; nliou@stust.edu.tw

**Keywords:** Jatropha curcas oil, non-isocyanate polyurethane, solvent resistant, chemical resistance, surface phenomena

## Abstract

Non-isocyanate polyurethane (NIPU) was prepared from Jatropha curcas oil (JCO) and its alkyd resin via curing with different diamines. The isocyanate-free approach is a green chemistry route, wherein carbon dioxide conversion plays a major role in NIPU preparation. Catalytic carbon dioxide fixation can be achieved through carbonation of epoxidized derivatives of JCO. In this study, 1,3-diaminopropane (DM) and isophorone diamine (IPDA) were used as curing agents separately. Cyclic carbonate conversion was catalyzed by tetrabutylammonium bromide. After epoxy conversion, carbonated JCO (CJCO) and carbonated alkyd resin (CC-AR) with carbonate contents of 24.9 and 20.2 wt %, respectively, were obtained. The molecular weight of CJCO and CC-AR were determined by gel permeation chromatography. JCO carbonates were cured with different amine contents. CJCO was blended with different weight ratios of CC-AR to improve its characteristics. The cured NIPU film was characterized by spectroscopic techniques, differential scanning calorimetry, and a universal testing machine. Field emission scanning electron microscopy was used to analyze the morphology of the NIPU film before and after solvent treatment. The solvent effects on the NIPU film interfacial surface were investigated with water, 30% ethanol, methyl ethyl ketone, 10% HCl, 10% NaCl, and 5% NaOH. NIPU based on CCJO and CC-AR (ratio of 1:3) with IPDA crosslink exhibits high glass transition temperature (44 °C), better solvent and chemical resistance, and Young’s modulus (680 MPa) compared with the blend crosslinked with DM. Thus, this study showed that the presence of CC-AR in CJCO-based NIPU can improve the thermomechanical and chemical resistance performance of the NIPU film via a green technology approach.

## 1. Introduction

Raw materials, the petroleum rate, resource depletion, and the awareness of the consumer society have increased worldwide attention on environmental issues and the growing concern for polyurethane (PU) materials. These issues have facilitated research on biodegradable and renewable green resources [1,2]. Compared with other renewable raw materials, vegetable oils (VOs) have attracted significant attention because of their low toxicity and environmental, regenerative, economic, and social advantages in the application of PU materials [1,3,4,5,6]. *Jatropha curcas* oil (JCO) is one of the attractive vegetable oils for the oleochemical industry [7,8,9,10,11]. JCO has high unsaturated fatty acid content and good oxidation stability [8], which make it a suitable raw material for many non-isocyanate polyurethane industrial applications [12] such as adhesives [13,14], alkyd epoxy resins [15], food packaging [16], surface coatings [17], nanocomposites [18], etc. Table 1 shows the characteristics of JCO compared to other type of vegetable oils. The low temperature viscosity (<25 °C) and acidic property of JCO are lower than those of soybean, linseed, and castor oils [19,20]. These properties increased the production of JCO as a biodiesel component and lubricant [21]. Different synthetic routes to modify JCO have been investigated to achieve this target. Epoxidation and transesterification have received increasing attention in recent years [21,22]. By contrast, CO_2_ transformation is a sustainable solution for controlling global warming and serves as a reusable resource for CO_2_ valorization.

The chemical fixation of CO_2_ into oxiranes, which produce five-member cyclic carbonates, has attracted economic and environmental attention. CO_2_ fixation into oxiranes is typically performed under various pressures and temperatures in the presence of the tetrabutylammonium bromide (TBAB) catalyst. TBAB is one of the most widely used homogenous catalysts that can decompose toxic and volatile compounds, such as hydrogen bromide, which is highly reactive with epoxy groups, at a high temperature [25]. However, other catalysts, such as silica-supported 4-pyrrolidinopyridium iodide, Pt-doped H_3_PW_12_O_40_/ZrO_2_, and KI with 18-crown-6 ether, have been tested. These catalysts exhibited low efficiency. The performance of TBAB has been enhanced by co-catalysts, such as CaCl_2_ and SnCl_4_·5H_2_O [26]. TBAB is used as a co-catalyst in the AlCl catalyst system for the CO_2_ fixation process [27]. The decomposition temperature of TBAB is a key factor in catalytic carbon dioxide fixation, and Zheng et al. determined that the decomposition temperature of TBAB is 130 °C [26].

The nature of the reactor system and its reaction time are interdependent. Tamami et al. [28] were the first to apply the flow of CO_2_ to synthesize carbonated soybean oil under atmospheric pressure at 110 °C using 5% of TBAB as a catalyst. They observed viscosity enhancement and a slow reaction rate during this reaction [29,30]. Mazo and Rios [30] reported improved carbonation kinetics. Doll and Erhan [25] intensified the carbonation process to achieve a high yield in a short time under supercritical CO_2_ and achieved 94% conversion in 20 h. The aminolysis of a five-member ring cyclic carbonate oligomer was intensified by the catalyst to produce efficient PU or PU-like materials under mild conditions [31]. This alternative pathway produces an isocyanate-free PU network and makes it stronger via β-hydroxyurethane linkages with promising chemistries [32].

Compared with conventional PU, ocyanate-free PU materials (i.e., non-isocyanate polyurethane (NIPU)) have lower permeability, better hydrolytic stability properties, and a safer fabrication process.

The formation of intramolecular hydrogen bonds via the hydroxyl group at the β-carbon atom in the PU chain enhances chemical resistance two times higher than the conventional PU [32]. NIPUs are insensitive to moisture in fillers or surfaces because of this unique molecular behavior. Surface interfacial phenomena are extremely versatile [33,34,35,36] and are a precise way to analyze the supramolecular chemistry of the material surface [37]. The surface properties depend on the surface charge developed by the functional moieties of the material, which are specific to the different segments of the NIPU network [33]. These segments establish different sorption processes with polarized molecular species in different solvent environments [38].

**Figure 1:**
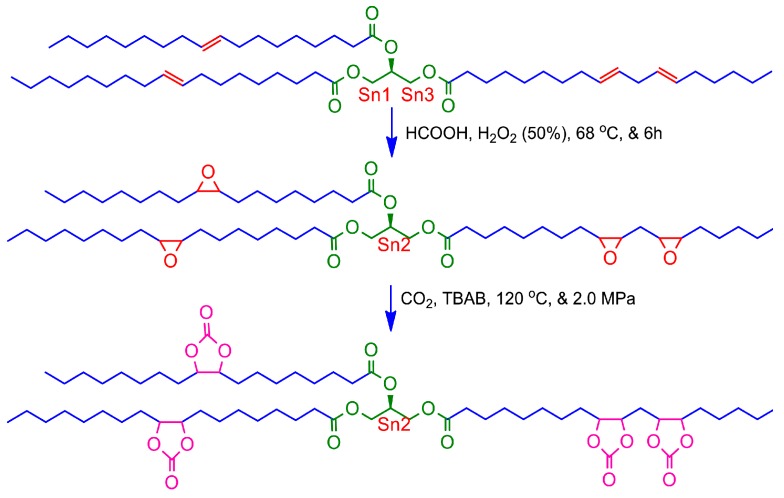
Schematic representation of catalytic carbon dioxide fixation via carbonation of epoxidized JCO (CJCO).

**Figure 2:**
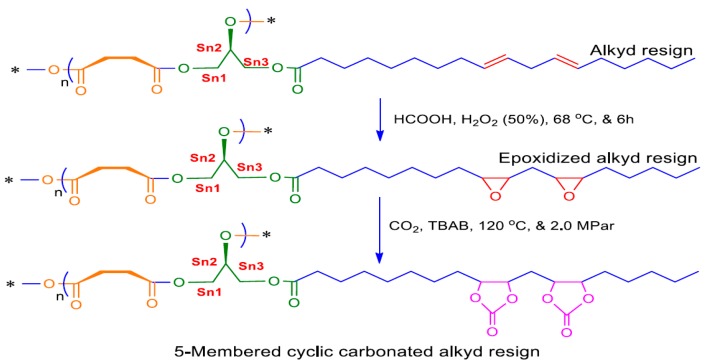
Reaction scheme of cyclic carbonation of epoxidized alkyd resin (CC-AR).

The major objective of this study is to optimize the reaction parameters, such as temperature and carbonation pressure, of epoxidized JCO, to assess the thermomechanical properties and the chemical resistance properties of cured JCO films and their blends based on NIPU. To the best of our knowledge, industrial JCO has not been previously investigated in NIPU production. Optimizing the cost-effective reaction parameters of CCJO is essential at the scale-up stage to facilitate mass production of JCO-based PU materials at the industrial level. Figure 1 illustrates the catalytic carbon dioxide fixation via carbonation of epoxidized JCO (CJCO). Figure 2 shows the reaction scheme of the cyclic carbonation of epoxidized alkyd resin (CC-AR). The isocyanate-free polymerization network was applied at different weight ratios of CCJO and CC-AR with 1,3-diaminopropane (DM) and isophorone diamine (IPDA). Thermomechanical, solvent, and chemical resistance properties were also investigated.

## 2. Materials and Methods

### 2.1. Materials

JCO was supplied by the Wahum Edible Oil Sdn. Bhd. (Cyberjaya, Malaysia). DM, ethyl acetate, acetic anhydrate, benzene, formic acid (HCOOH; 85%), hydrogen peroxide (H_2_O_2_; 50%), H_2_SO_4_, pyridine, CaO, HCl, NaCl, NaOH, TBAB, IPDA, CO_2_, methyl ethyl ketone (MEK), perchloric acid, denatured ethanol, KOH, phenolphthalein, sodium bicarbonate, anhydrous sodium sulfate, triethylamine, and tetrahydrofuran were purchased from Sigma-Aldrich (Kuala Lumpur, Malaysia). All materials were used as received without any further purification.

### 2.2. Synthesis and Characterization of JCO Based Cyclic Carbonates

CJCO is obtained through a two-step synthesis, as shown in Figure 1. The first step involves the epoxidation of JCO with H_2_O_2_ in the presence of formic acid. The second step involves catalytic carbon dioxide fixation through carbonation of the epoxidized JCO (EJCO).

#### 2.2.1. Preparation of EJCO

JCO epoxidation was conducted in a three-necked round-bottom flask that contains formic acid (4.6 g) with JCO (100 g). Hydrogen peroxide (115.6 g, 50% (*w*/*v*)) was added to the flask with continuous stirring. The reaction mixture was maintained at 65 °C for 6 h. The resulting mixture was washed with distilled water, followed by sodium bicarbonate solution until it reached a neutral pH. The oily phase was collected and dried with anhydrous sodium sulfate.

An ultrasonication-assisted titration method was used to determine the epoxy value (Equation (1)). The epoxy conversion was then calculated using Equation (2).

(1) $$\mathrm{Epoxy}\;\mathrm{value}\;\left(\mathrm{EV}\right)=\frac{\left(V_{0}-V\right)\times N}{W\times 10}$$

(2) $$Epoxy\;conversion\;\left(EC\right)=\frac{EV}{W}\times 100\%$$

where *V*_0_ is a required volume of the NaOH solution (mL) for blank titration; *V* is a required volume of the NaOH solution (mL) to titrate the hydrochloric acid-acetone solution with dissolved sample; *N* = normality of NaOH standard aqueous solution and W is the mass (g) of the sample used for the titration.

#### 2.2.2. Preparation of CJCO

Catalytic carbon dioxide fixation via the carbonation of EJCO was conducted in the presence of TBAB using a CO_2_ pressure reactor. EJCO (100 g) was transferred into the reactor. Then, carbon dioxide and TBAB (3.5 g) were added and heated to 120 °C. Complete conversion was monitored by Fourier transformed infrared (FTIR) spectroscopy, proton nuclear magnetic resonance (^1^H-NMR) spectroscopy, and titration measurements. Epoxy conversion was measured at different temperatures and pressures that range from 110 to 140 °C and from 1.0 to 2.0 MPa, respectively.

The carbonated content of the purified sample was determined from characteristic peaks of cyclic carbonate using the equations given below as described previously by Cornille et al. [39]. A standard solution of DMSO with toluene (60 mg of toluene in 10 mL of DMSO-*d*_6_) and a specific amount of cyclic carbonate (around 30 mg) were weighted and then put into an NMR tube for carbonate content determination.

(3) $$\mathrm{CEW}=\frac{m\times I_{{\mathrm{CH}}_{3}}}{I_{\mathrm{CC}}\times n_{\mathrm{toluene}}}$$

(4) $$\mathrm{Carbonate}\;\mathrm{content}=\frac{\mathrm{CEW}}{m}\times \mathrm{EC}\times 100\%$$

where $I_{\mathrm{CC}}$ is the total integral area of the characteristic peaks of cyclic carbonate; and $I_{{\mathrm{CH}}_{3}}$ is the integral area of the characteristic peak of the –CH_3_ group of the toluene in the ^1^H-NMR spectra; $n_{\mathrm{toluene}}$ is the molar amount of toluene introduced in the standard solution; CEW is a carbonate equivalent weight and m is the mass of the sample introduced in the ^1^H-NMR tube.

#### 2.2.3. Optimization of the Reaction Parameters of CJCO

The conversion yield of EJCO to CJCO was determined within the temperature range of 100 °C to 140 °C and pressure range of 1.0 to 2.0 MPa with a constant amount of TBAB. The yield of the catalytic carbon dioxide fixation via carbonation of EJCO was monitored by FTIR and ^1^H-NMR spectroscopy. The carbonation yield increased with the increase in temperature with the Arrhenius law. Moreover, the mass transfer kinetics increased with the increase in reaction temperature [40]. 

**Figure 3:**
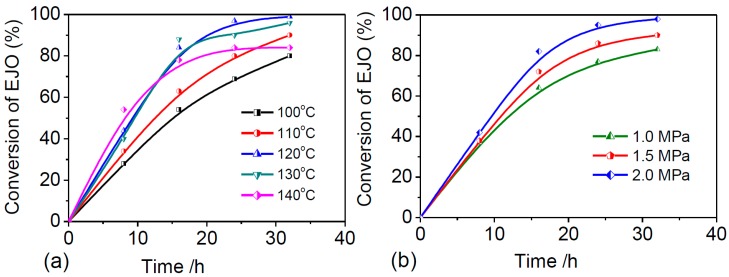
The effect of reaction parameters on the carbonation yield of EJCO at a fixed amount of 3.5 g of TBAB (**a**) Influence of temperature at 2.0 MPa and (**b**) Influence of pressure at 120 °C.

In the present study, high reaction temperatures of 130 to 140 °C exhibited low conversion yield of five-member cyclic carbonate. However, a high yield was achieved at 120 °C under 2.0 MPa, as shown in Figure 3a. This finding could be attributed to the thermal decomposition and mass loss of TBAB, which significantly influences the catalytic carbon dioxide fixation [41]. Figure 3b shows no significant differences in the initial carbonation yield between 1.0 and 2.0 MPa of CO_2_. However, the carbonation yield of EJCO varied after 30 h. Furthermore, Motokura et al. [42] have observed certain advantages in increasing the CO_2_ pressure between 3.0 and 5.0 MPa at 130 °C within a 7 h reaction period. In this study, the high carbonation period of 30 h and the stability of TBAB at low temperature were linked to a high CO_2_ gradient. Figure 5 shows the high carbonation yield at a low reaction pressure of 2.0 MPa and low temperature of 120 °C. Considering the thermal stability of TBAB and the carbonation yield with respect to the CO_2_ pressure, it could be stated that the optimal carbonation conditions would occur at the temperature of 120 °C and under the pressure of 2.0 MPa of CO_2_ with a fixed amount of 3.5 mol % of TBAB. Table 2 shows the comparative reaction parameters used for cyclic carbonation of various VOs in the presence of TBAB.

### 2.3. Synthesis of JCO-Based Catalytic Carbonated Alkyd Resin

#### 2.3.1. Preparations of JCO-Based Monoglyceride Alkyd Resin

A mixture of glycerol and JCO was placed in a three-necked round-bottom flask connected to a reflux tube, a N_2_ gas inlet, and a thermometer. The flask was immersed in the oil bath placed in a magnetic stirrer. The reaction was maintained at 230 °C for 1.5 h in the presence of 0.03 wt % CaO and was continued until a drop of reaction mixture could be completely dissolved by the methanol. (The drop of reaction mixture was cooled down first to 40 °C at normal atmosphere before adding into methanol). The reaction mixture was then cooled to 120 °C and 0.12 mol of succinic anhydride was added to the mixture. The temperature of the mixture was increased to 240 °C and was maintained until an acid value in the range of 10–15 was obtained. The synthezised alkyd resin was used for the preparation of EAR and CC-AR in the following steps. 

#### 2.3.2. Preparation of Epoxidation and Cyclic Carbonation of JCO-Based Alkyd Resin (CC-AR)

The epoxidation and cyclic carbonation of alkyd resin were conducted by following the same procedures as that of the functional group modification of JCO, as shown in Figure 2. The synthesis method of CC-AR was similar to the synthesis of CJCO. Since the optimization has been done on the CJCO synthesis, the same optimization parameters were also used for the preparation of CC-AR. 

### 2.4. Synthesis and Characterization of CJCO and Its Blends Based on NIPU

JCO-based non-isocyanate polyurethane (NIPU) was prepared using the different compositions of CJCO and CC-AR in the presence of trimethylamine. The calculated amounts of CJCO, CC-AR, and diamine were transferred into a three-necked round-bottom flask that contained MEK. The feed weight of amine was determined based on the mole ratio of amine to carbonate. The reaction mixture was heated to 70 °C for 8 h and to 100 °C for 10 h with continuous stirring at 600 rpm to make NIPU with IPDA, as described previously [44]. Meanwhile, NIPU with DM was confirmed at 1 h by FTIR spectroscopy. FTIR spectroscopy was used to monitor the progress of the reaction. Then, the resulting mixture was placed into a 55 °C oven overnight to remove moisture and trapped air. After the removal of excess water and trapped air, the mixture was poured onto a Teflon sheet using an applicator. The curing procedure was conducted at 70 °C for 10 h, followed by 120 °C for 4 h.

### 2.5. Characterization Study

#### 2.5.1. Molecular Weight

The molecular weight distribution of the samples was obtained by gel permeation chromatography (GPC-THF system) at 40 °C in the 510 pump with a flow rate of 1 mL·min^−1^.

#### 2.5.2. Structural Characterization

FTIR spectroscopy was conducted to analyze the functional groups of the samples and cured NIPU film using the PerkinElmer Spectrum 400 FTIR spectrometer unit (PerkinElmer, Waltham, MA, USA)using the KBr pellet technique with the resolution of 4 cm^−1^ and 32 scans per recording. ^1^H-NMR spectroscopy was performed on a Bruker ARX 300 spectrometer (Bruker Biospin, Switzerland) operating at 600 Hz to study the chemical shifts of the protons and carbonate content using CDCl_3_ as a solvent, and chemical shifts (*δ*) were reported in parts per million. The surface morphology of the NIPU film was investigated using a field emission scanning electron microscope (FESEM, ZEISS Sigma 500, Jena, Germany) before and after solvent and chemical treatments.

#### 2.5.3. Thermo-Mechanical Properties

Differential scanning calorimetry (DSC) was used to determine the glass transition temperature *T*_g_, wherein 10 mg samples were heated on the TA DSC Q200 calorimeter (TA instrument, New Castle, DE, USA) from 10 to 250 °C at a heating rate of 10 °C·min^−1^. The ultimate tensile strength, Young’s modulus (*E*), and percent elongation at break (*ε*_max_) of the blends were obtained by a universal testing machine (Shimadzu AGS-X series, Tokyo, Japan) at room temperature. Five test specimens were used to obtain the mean value of all tests. The effect of carbonated alkyd resin on the thermomechanical properties of CJCO and its blends based on NIPU was investigated with respect to the mixing ratio of CJCO and CC-AR and the amine content.

#### 2.5.4. Solvent and Chemical Resistance Tests

Solvent and chemical resistance tests were conducted in different solvent environments. Water, ethanol (30% aq.), MEK, NaOH, HCl, and NaCl were used for this purpose.

### 2.6. Characterization of EJCO and CJCO

**Figure 4:**
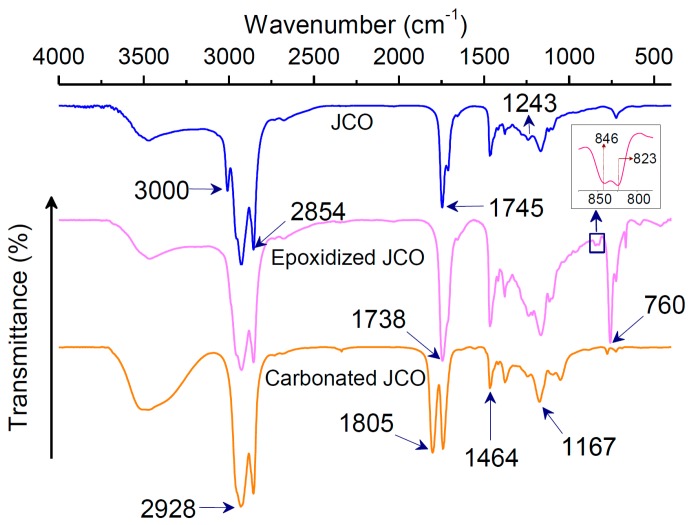
FTIR spectra of Jatropha curcas oil (JCO), epoxidized JCO, and carbonated JCO.

**Figure 5:**
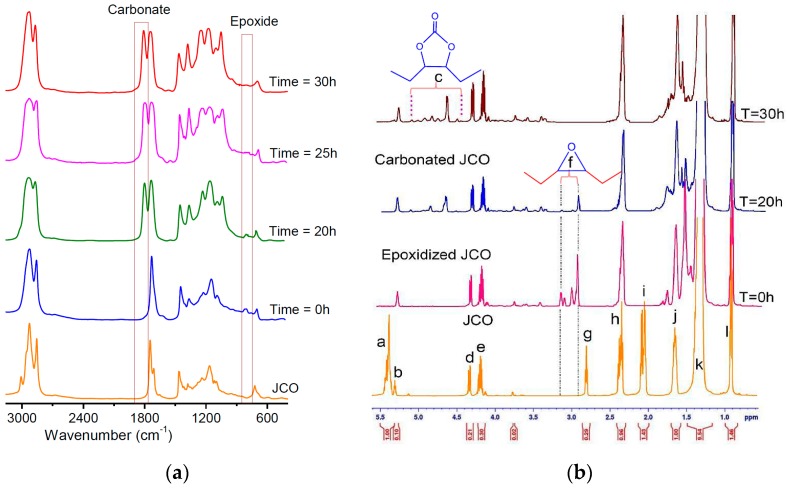
The spectra of (**a**) FTIR: (**b**) ^1^H-NMR at different reaction times at 120 °C, 2.0 MPa, and 3.5 mol % of TBAB.

JCO was received as golden yellow oil, and the functional moieties of JCO were investigated by FTIR spectroscopy, as illustrated in Figure 4. The respective peaks were observed at 2926 cm^−1^ (–C–H symmetric stretching vibration of –CH_2_ groups), 2854 cm^−1^ (–C–H asymmetric stretching vibration of –CH_2_ groups), 1745 cm^−1^ (C=O stretching vibration), 1652 cm^−1^ (assigned to the –CH=CH–), 1464 cm^−1^ (–CH_2_ scissoring), 1243 cm^−1^ (–CH_2_ wagging), 1167 cm^−1^ (–C–O stretching of ester), and the peak at 760 cm^−1^ is attributed to –CH_2_ rocking vibration. The chemical shift of the JCO protons was examined by ^1^H-NMR as shown in Figure 5b, with the peaks between 5.5 and 5.32 (**a**, –CH=CH–, 8H), 5.32–5.25 (**b**, CH–O–C=O, 1H, attributed to the sn-2 glycerol proton), 4.34–4.13 (**d** and **e**, CH_2_–O–C=O, 4H, sn-1,3 glycerol protons), 2.85–2.75 (**g**, CH=CH–CH_2_–CH=CH, 2H, bisallylic group of C18:2), 2.4–2.3 (**h**,–CH_2_–C=O, 6H), 2.1–2.0 (**I**, –CH_2_–CH=CH–, 12H, allylic –CH_2_ group of C18:2 and C18:1), 1.69–1.58 (**j**, β–CH_2_–, 6H), 1.41–1.24 (**k**, –CH_2_–of fatty acid), and 0.93–0.88 (**l**, terminal CH_3_ of fatty acid, 9H, sn-1, sn-2, sn-3 are stereospecific acyl carbons [45]). 

EJCO was obtained as a clear pale yellow viscous product. FTIR spectroscopy confirmed the epoxy ring formation with peaks at 846 and 823 cm^−1^, as shown in Figure 4 and Figure 5a. ^1^H-NMR spectroscopy exhibited the chemical shift of the EJCO protons, as illustrated in Figure 5b, with peaks between 5.30 and 5.25 (**a**, CH–O–C=O, 1H, attributed to *sn*-2 glycerol proton) and between 4.36 and 4.12 (**d** and **e**, CH_2_–O–C=O, 4H, *sn*-1,3 glycerol protons), 3.16–3.08 (**f**, –CHOCH–CH_2_– CHOCH–, diepoxides of linoleic acid), 3.02–2.96 (**f**, –CHOCH–CH_2_–CHOCH–, diepoxides of linoleic acid), 2.96–2.89 (**f**, –CH_2_–CHOCH–CH_2_–, epoxides of linoleic acid and its overlap with the epoxy oleic acid), 2.4–2.3 (**h**, –CH_2_–C=O, 6H), 1.69–1.58 (**j**, *β*–CH_2_–, 6H), 1.41–1.24 (**k**,–CH_2_–of fatty acid), and 0.93–0.88 (**l**, terminal CH_3_ of fatty acid, 9H, *sn*-1, *sn*-2, *sn*-3 are stereospecific acyl carbons [23]).

The carbonate content of triglyceride (JCO) and alkyd resin was calculated using the initial and final epoxy contents after the complete removal of TBAB. Ethyl acetate was used to separate TBAB from carbonated JCO. CJCO was obtained as a clear wine red viscous product. The peak at 1805 cm^−1^ of the FTIR spectra confirmed the formation of the five-member cyclic carbonate, as illustrated in Figure 4. The chemical shift of the CJCO protons was examined by ^1^H-NMR spectroscopy, as shown in Figure 5b. The corresponding peaks were observed between 5.30 and 5.25 (a, CH–O–C=O, 1H, *sn*-2 glycerol proton), 5.06–4.52 (c, –CHOCOOCH–CH_2_–CHOCOOCH–, carbonation of linoleic acid), 4.36–4.12 (d and e, CH_2_–O–C=O, 4H, *sn*-1,3 glycerol protons), 2.96–2.89 (h, –CH_2_–CHOCH–CH_2_–, the partial epoxides of linoleic acid and its overlap with the epoxy oleic acid), 2.4–2.3 (i, –CH_2_–C=O, 6H), 1.69–1.58 (j, *β*–CH_2_–, 6H), 1.41–1.24 (k, –CH_2_–of fatty acid), and 0.93–0.88 (l, attributed to the terminal CH_3_ of the fatty acid, 9H). 

## 3. Results and Discussion

### 3.1. Characterization of Monoglyceride, Alkyd Resin (AR), Epoxidized (EAR), and CC-AR

**Scheme 1:**
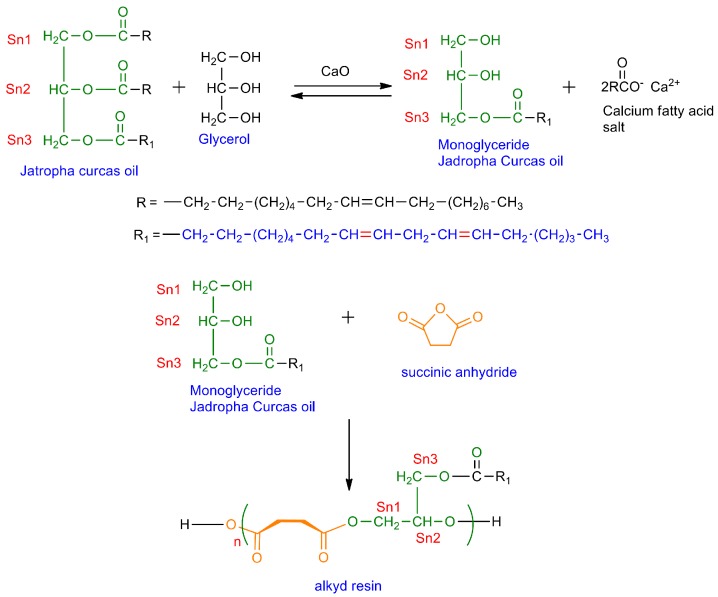
Reaction scheme of monoglyceride and alkyd resin of the *Jatropha* *curcas* oil.

**Figure 6:**
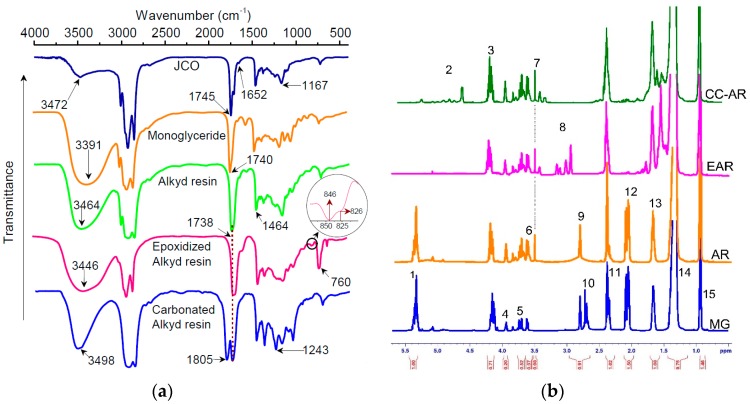
Spectra of catalytic carbon dioxide fixation via carbonation of epoxidized JCO based alkyd resin (**a**) FTIR spectra; (**b**) ^1^H-NMR with reaction parameters of 120 °C, 2.0 MPa, and 3.5 mol % of TBAB.

FTIR and ^1^H-NMR analyses were applied to examine the corresponding peak confirmation and chemical shift of the monoglyceride, AR, EAR, and CC-AR protons. Scheme 1 illustrates the reaction scheme of the monoglyceride and AR synthesis. The FTIR spectrum of monoglyceride assigns the peaks at 3391 cm^−1^ (attributed to the hydroxyl groups of the monoglyceride of JCO) and 2855 cm^−1^ (–C–H asymmetric stretching vibration of –CH_2_ groups), 3464 cm^−1^ (attributed to the hydroxyl groups of the alkyd resin of JCO), 1740 cm^−1^ (C=O stretching vibration of monoglyceride), 1738 cm^−1^ (C=O stretching vibration of alkyd resin), 1652 cm^−1^ (assigned to the –CH=CH–), 1464 cm^−1^ (–CH_2_ scissoring), and 1243 cm^−1^ (–CH_2_ wagging), as illustrated in Figure 6a.

Figure 6b shows the ^1^H-NMR spectrum of monoglyceride, wherein the corresponding chemical shift of the protons was observed between 5.5–5.32 (**1**, –CH=CH–, 2H), 4.20–4.13 (**3**, CH_2_–O–C=O, 2H, *sn*-3 glycerol protons), 3.98–3.92 (**4**, CH_2_O–CO–CH_2_–, 2H), 3.83–3.80 (**5**, –CHOH–, 1H), 3.64–3.59 (**6**, –CH_2_OH–, 4H), 2.81–2.77 (**9**, –CH=CH–CH_2_–CH=CH–, 2H, bisallylic group of C18:2), 2.72–2.67 (**10**, –CH_2_OH– and –CHOH–, 2H), 2.4–2.3 (**11**, –CH_2_–C=O, 6H), 2.1–2.0 (**12**, –CH_2_–CH=CH–, 12H, allylic –CH_2_ group of C18:2 and C18:1), 1.69–1.58 (**13**, *β*–CH_2_–, 6H), 1.41–1.24 (**14**, –CH_2_–of fatty acid), and 0.93–0.88 (**15**, attributed to the terminal CH_3_ of the fatty acid, 9H, *sn*-3 is stereospecific acyl carbons [23]). Figure 6b also shows the chemical shift of the AR protons, wherein the peaks were observed between 5.5–5.32 (**1**, –CH=CH–, 2H), 4.34–4.13 (**3**, CH_2_–O–C=O, 2H, *sn*-3 glycerol protons), 3.98–3.92 (**4**, CH_2_O–CO–CH_2_–, 2H), 3.83–3.80 (**5**, –CHOH–, 1H), 3.64–3.59 (**6**, –CH_2_OH–, 4H), 3.48 (**7**, –CH_2_OH–, 1H), 2.81–2.77 (**9**, –CH=CH–CH_2_–CH=CH–, 2H, bisallylic group of C18:2), 2.4–2.3 (**11**, –CH_2_–C=O, 6H), 2.1–2.0 (**12**, –CH_2_–CH=CH–, 12H, allylic –CH_2_ group of C18:2 and C18:1), 1.69–1.58 (**13**, *β*–CH_2_–, 6H), 1.41–1.24 (**14**, –CH_2_–of fatty acid), and 0.93–0.88 (**15**, attributed to the terminal CH_3_ of the fatty acid, 9H [23]).

The functional groups of EAR and the CC-AR were confirmed by FTIR spectroscopy, as illustrated in Figure 6a, where the corresponding peaks were observed at 846 and 826 cm^−1^ (attributed to epoxy ring formation), 3446 cm^−1^ (attributed to the hydroxyl groups of EJCO), 2855 cm^−1^ (–C–H asymmetric stretching vibration of –CH_2_ groups), 3498 cm^−1^ (attributed to the hydroxyl groups of CJCO), 1805 cm^−1^ (C=O attributed to the stretching vibration of carbonyl), 1738 cm^−1^ (C=O stretching vibration of alkyd resin), 1652 cm^−1^ (assigned to –CH=CH–), 1464 cm^−1^ (attributed to –CH_2_ scissoring), and 1243 cm^−1^ (attributed to–CH_2_ wagging). The ^1^H-NMR spectrum exhibits the chemical shift of the EAR and CC-AR protons (Figure 6b). The peaks were observed between 3.16–3.08 (**8**, –CHOCH–CH_2_– CHOCH–, diepoxides of linoleic acid), 3.02–2.96 (**8**, –CHOCH–CH_2_–CHOCH–, diepoxides of linoleic acid), and 2.4–2.3 (**11**, –CH_2_–C=O, 6H), 1.69–1.58 (**13**, *β*–CH_2_–, 6H), 1.41–1.24 (**14**, –CH_2_–of fatty acid), and 0.93–0.88 (**15**, attributed to the terminal CH_3_ of the fatty acid, 9H), respectively. The observed peaks of CC-AR between 5.06–4.52 (**2**, –CHOCOOCH–CH_2_–CHOCOOCH–, attributed to the carbonation of linoleic acid), 4.36–4.12 (**3**, CH_2_–O–C=O, 4H, *sn*-3 glycerol protons), and 2.4–2.3 (**11**, –CH_2_–C=O, 6H) confirmed the five membered cyclic carbonate fixation of EAR, as illustrated in Figure 2. 

### 3.2. Characterization of CJCO and CC-AR

The wide use of TBAB is attributed to its faster epoxy conversion than that of heterogeneous catalysts [42]. The present study set the reaction temperature to 120 °C to synthesize CJCO and CC-AR with approximately 100% epoxy conversion at 2.0 MPa in the presence of TBAB for NIPU preparation. The initial molecular weight of the JCO was 900 g·mol^−1^ which is greater than the normal vegetable oil (triglyceride, 800 g·mol^−1^). After chemical modification of JCO to CJCO, the molecular weight of JCO increased from 900 g·mol^−1^ to 1684 g·mol^−1^. This is because the higher number of unsaturated double bonds in JCO has been transferred to the cyclic carbonate functional groups in the triglyceride molecules of CJCO [8]. CC-AR demonstrated high viscosity of 3400 ± 520 MPa·s, which was higher than that of CJCO at 25 °C (2900 ± 430 MPa·s) after the removal of TBAB. The number of linear fatty acid chains of the alkyd resulted in the low amount of carbonate in CJCO and CC-AR, as shown in Table 3.

### 3.3. Thermo-Mechanical Properties of the NIPU Film

**Scheme 2:**
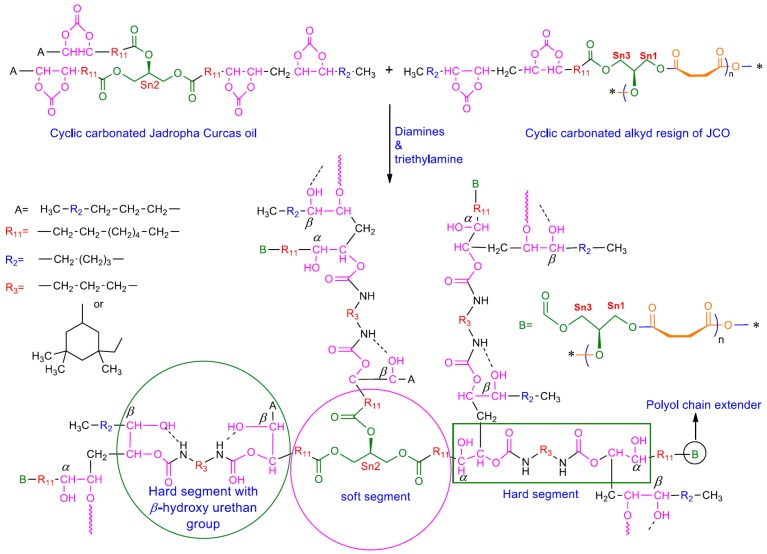
The crosslinked polymer network of NIPU with β-hydroxyurethane links.

**Figure 7:**
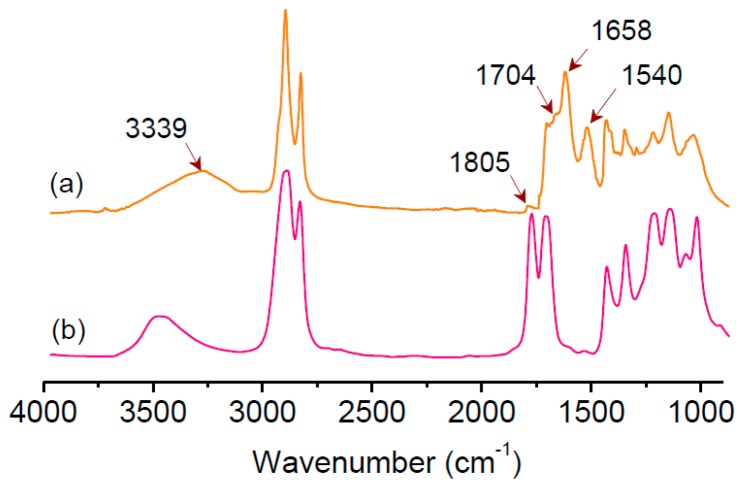
FTIR spectra of CJCO (**a**) after being cured with isophorone diamine (IPDA); (**b**) before curing with isophorone diamine.

Scheme 2 illustrates the crosslinked polymer network of NIPU with β-hydroxyurethane links. FTIR spectroscopy was used to investigate the formation of the urethane bond, as illustrated in Figure 7. The spectrum of the NIPU film assigns the peaks at 3339 cm^−1^ (attributed to the –N–H– vibration of NIPU), 1704 cm^−1^ (attributed to the C–O stretching vibration of urethane), 1658 cm^−1^ (attributed to the C–O–C stretching vibration of NIPU), and 1540 cm^−1^ (assigned to the N–H– bending and C–N– vibration of NIPU).

Moreover, the disappearance of the corresponding carbonyl peak at 1805 cm^−1^ and the hydroxyl group at 3464 and 3446 cm^−1^ of CJCO and CC-AR confirmed the formation of the urethane group via the complete conversion of NIPU [35]. In this study, there was no gel fraction that was found. A complete film formation occurred after a full curing reaction with diamines. This was confirmed in our FTIR study that after the curing reaction with IPDA, a complete urethane characteristic peak was observed as illustrated in Figure 7.

The thermomechanical properties of the prepared CJCO and their blend-based NIPUs are listed in Table 4. These blends were cured with IPDA and DM, wherein CC-AR weight and amine content were used as functions to investigate their properties. Mechanical properties, such as Young’s modulus, elongation at break, and tensile strength, were effectively changed with the CC-AR weight and amine content. The CJCO-based film cured with DM exhibited high elongation at break (230%), but low modulus. However, the CJCO/CC-AR blend with a ratio of 1:3 showed significant improvement of Young’s modulus of 680 MPa with the curing agent of IPDA. This finding can be attributed to steric hindrance of the cycloaliphatic content of IPDA [46]. This finding might also be due to the structural rigidity of NIPU resulting from the highly crosslinked network structure [47]. The CJCO/CC-AR blend (ratio of 1:3) exhibits approximately a three times higher Young’s modulus (680 MPa) and higher *T*_g_ value (44 °C) with IPDA compared with DM, as shown in Table 4. The CJCO and CJCO/CC-AR blend crosslinked with IPDA show higher *T*_g_ compared to those which were crosslinked with DM due to the presence of the cycloaliphetic content in the IPDA material. In this study, the trend of *T*_g_ of the CJCO/CC-AR blends crosslinked with DM was not significant. This insignificant trend of *T*_g_ might be due to slippage between the chains of the saturated polymer matrix and the excess amount of CC-AR. The blends of CJCO/CC-AR crosslinked with IPDA show a significant drop of *T*_g_ with the increase of CC-AR composition due to the amorphous nature of CC-AR [47]. 

This finding can be attributed to the uniform and stoichiometric distribution of the high and low five-member cyclic carbonate functionalities of CJCO and CC-AR, which enhanced the crosslink density of the polymerization network [44]. However, the blend of CJCO/CC-AR with high CC-AR composition (ratio of 1:4) shows a reduction in modulus and tensile strength properties compared with the NIPU-based blend with CC-AR in the ratio of 1:3 (*w*/*w*). It is believed that the saturation and uniform distribution has been achieved in the CJCO/CC-AR blend with the blend ratio of 1:3. Thus, the further increase of CC-AR in the CJCO/CC-AR blend with the ratio of 1:4 (*w*/*w*) would result in slipping between the chains of the saturated polymer matrix [48]. Thus, the excess amount of CC-AR would jeopardize the mechanical properties of the resulting NIPU.

### 3.4. The Solvent Effect on the Surface Interfacial Phenomena

The solvent and chemical resistant properties of the NIPU films were measured by the swelling test. The effect of different solvents on the interfacial surface of the NIPU film was investigated based on the attractive forces caused by the polarity of the film surface and the solvent systems [36]. On one hand, the residual attractive forces are generated by the surface active functional groups, such as urethane, β-hydroxyl, ester, and alkyl groups of the hard, soft, and polyol chain extender segments of the NIPU film surface [49]. Consequently, these forces can establish a tendency to attract and retain the active species of the solvent systems when they are in contact with the film surface [50]. On the other hand, these active species, such as molecules, solvated cation, or anions, of the different solvent systems have attracted the surface active functional groups of the solid surface via sorption processes [34,35]. These sorption processes could be either negative or positive, where the negative sorption process decreases the rate of swelling while weight augmentation could result in a positive sorption process. However, the effect of different solvents on the interfacial surface of the NIPU film can be enhanced only by chemisorption and/or physisorption processes [35,36,37].

#### 3.4.1. Solvent Resistance and Surface Interfacial Phenomena of NIPU

**Figure 8:**
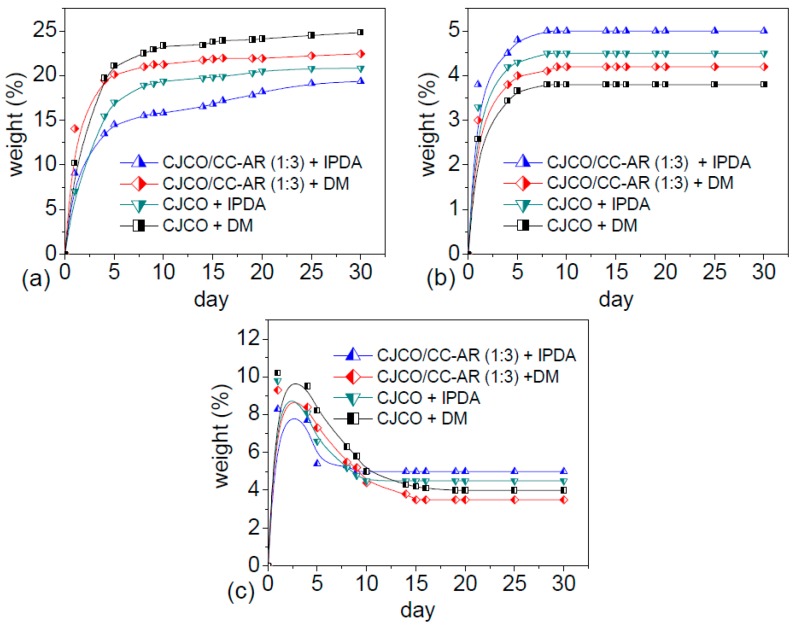
Swelling-time diagrams of CJCO and CJCO/CC-AR blends in (**a**) water; (**b**) solvent 30% ethanol; (**c**) solvent methyl ethyl ketone.

Figure 8 illustrates the solvent resistance of the NIPU film via interfacial phenomena. The weight percentage of the NIPU film was varied with respect to the amine content and steric hindrance of the alkyl groups of the NIPU film [46]. Figure 8a shows the swelling–time diagram of CJCO and its blend with CC-AR-based NIPU in water. 

Water adsorption on the NIPU film surface was initiated, followed by chemisorption and physisorption via cohesive interaction (intermolecular forces) of the water molecules [49,51]. These sorption processes could be enhanced by the polarity of the functional moieties of the film surface, particularly the β-hydroxyurethane groups of the hard segments. Consequently, the imbalanced residual attractive force of the NIPU film surface initiated the chemisorption process. Moreover, the interfacial surface of the NIPU film produced the monolayer via polar forces and acid–base interactions [37]. However, a lower rate of adsorption was observed in the CJCO/CC-AR blend (ratio of 1:3) with IPDA compared with the CJCO/CC-AR blend (ratio of 1:3) with DM (Figure 8a). 

This phenomenon might occur due to the high steric hindrance of the cycloaliphatic content of the IPDA. The swelling behavior of the NIPU films was significantly reduced in the ethanol–water system (Figure 8b) compared with that in water (Figure 8a). This finding may be due to the strong dipole–dipole interactions of ethanol with hydronium ions in the ethanol–water system [51], which can lead to the increase of strong cohesive attraction. Ethanol is an electro-active species [52]. When ethanol is added into water, ethanol molecules like to stay in the solution due to strong interactions between the ethanol and water which reduces the rate of swelling [53]. 

**Figure 9:**
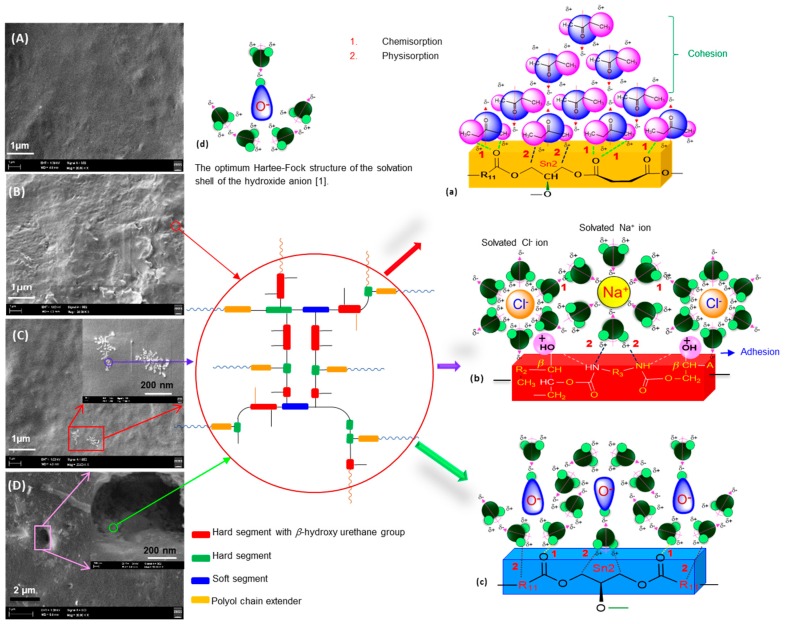
FESEM images of the CJCO/CC-AR blend (ratio 1:3) based NIPU with IPDA: (**A**) before solvent treatment; (**B**), (**C**), and (**D**) after solvent treatment. Interfacial surface of the NIPU: (**a**) after being treated with MEK; (**b**) after being treated with 10% NaCl; (**c**) after being treated with 5% NaOH; (**d**) the optimum Hartee-Fock structure of the solvation shell of the hydroxide anion [1].

However, the adhesive attractions of the polar ethanol molecules might be enhanced by the β-hydroxyurethane groups of the NIPU film surface via weak van der Waals forces [27] toward the film surface in the ethanol–water system. Consequently, Figure 8b shows that the CJCO/CC-AR blend (ratio of 1:3) with IPDA exhibited a higher swelling rate than the CJCO/CC-AR blend (ratio of 1:3) with DM. By contrast, a slight weight loss was observed in the swelling–time diagram of the NIPU film under the MEK solvent, as shown in Figure 8c. This phenomenon could be due to the nucleophilic attraction of the ester groups on the interfacial surface [40] of the NIPU film, as illustrated in Figure 9a. This attraction might be due to steric hindrance of the alkyl groups on the film surface, which generated a strong electrostatic interaction toward the MEK molecules. Consequently, the chemisorption process between the MEK molecules and interfacial surface of the NIPU film resulted in polyol chain extender segments. 

Moreover, the polarized MEK altered the morphology of the film, as shown in Figure 9B compared with Figure 9A. This alteration may be due to the heterolytic cleavage of the polymeric chains on the interfacial surface of the NIPU film. The strong intermolecular interactions between polymer chains were established by low steric hindrance [41,42]. Furthermore, the steric hindrance of the alkyl groups on the film surface might be reduced via electrophilic attractions on the inner surface of the β-hydroxyurethane groups of the NIPU films. Consequently, the swelling behavior remains constant and differs from one another with respect to the amine content of the NIPU films.

#### 3.4.2. Chemical Resistance and Surface Interfacial Phenomena of NIPU

**Figure 10:**
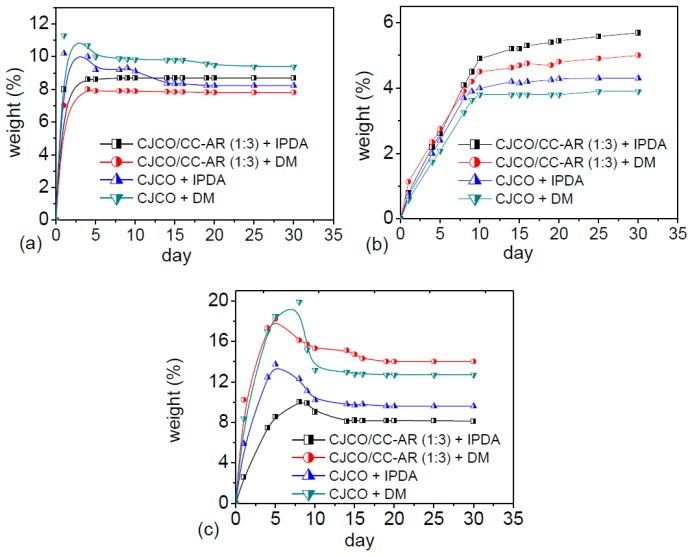
Swelling-time diagrams of CJCO and CJCO/CC-AR blends in (**a**) dilute 10% HCl solution; (**b**) 10% NaCl solution; (**c**) 5% NaOH solution.

The chemical resistance property of the NIPU films was investigated in different chemical environments, such as diluted HCl (10%), aqueous NaCl (10%), and NaOH (5%). The carbonate content, crosslinking density, β-hydroxyl moiety, and intermolecular rigidity of the blends show a significant effect on the chemical resistance behavior of the cured films. Moreover, the soft segments contribute to alteration of the film surface by chemisorption, whereas the hard segments enhance physisorption via β-hydroxyurethane hydrogen bonds. Figure 10a illustrates the swelling property of the NIPU films in diluted HCl solution where the CJCO-based films with DM and IPDA exhibited a slight weight loss. The strong nucleophilic attraction of the ester groups of the polyol chain extender segments toward the hydronium ions of the solvent could patronize this phenomenon [54,55] as shown in Figure 9b. However, the cohesive attraction between the solvated chloride ions (SCI) and the hydronium ions ensures that the weight of the film remains constant [51].

By contrast, all NIPU films exhibited a significant weight augmentation in their swelling–time diagram under the NaCl solvent system, as shown in Figure 10b. Figure 9C shows the morphology of the film of the CJCO/CC-AR blend (ratio of 1:3) cured with IPDA after salt treatment. The weight augmentation might be due to the strong electrostatic interactions of the SCI toward the β-hydroxyurethane groups on the film surface, which could promote chemisorption [56].

The weight augmentation might also be due to the electrophilic attraction of the solvated Na^+^ ions toward SCI [27]. The optimum Hartree–Fock structure of the solvation shell of the hydroxide anion in the OH–(H_2_O)_17_ cluster presents four water molecules coordinated to the ^−^OH oxygen and one water molecule coordinated to hydrogen, thus making the total solvation number of ^−^OH in this cluster equal to 5 [57,58], as shown in Figure 9d. Consequently, Figure 10c illustrates the significant effect of the polarized solvation shell of the hydroxide anions on the swelling property of the NIPU films. This phenomenon reflects the strong chemisorption and physisorption via electrophilic attractions of the anions toward the ester groups and alkyl chains of the soft segments, as illustrated in Figure 9c. Consequently, the CJCO/CC-AR blend (ratio of 1:3) with IPDA exhibited a slight weight loss compared with the CJCO/CC-AR blend (ratio of 1:3) with DM. This phenomenon reflects the strong chemisorption and physisorption via electrophilic attractions of the anions toward the ester groups and alkyl chains of the soft segments, as illustrated in Figure 9c. Figure 9D shows that the polarized solvation shell of the hydroxide anions takes part in heterolytic cleavage of the NIPU film surface [59].

## 4. Conclusions

In this study, the carbonation of EJCO and monoglyceride-based carbonated alkyd resin was conducted successfully. The conditions for epoxidation and carbonation reactions will significantly influence the carbonate content of CJCO and CC-AR. The optimal reaction condition was set at 120 °C and 2.0 MPa of CO_2_ pressure. The carbonate conversion of CJCO and CC-AR was observed at 99% and 97%, respectively, within a 30 h reaction period. The carbonate content of CJCO was higher than that of CC-AR. Compared with the CJCO-based NIPU, the blend of CJCO and CC-AR-based NIPU exhibits excellent thermal, mechanical, solvent, and chemical resistance properties when cured with DM and IPDA. Furthermore, the amine and cycloaliphatic contents of IPDA play a major role in surface interfacial phenomena via sorption processes. In the present study, the strong solvent and chemical resistance properties exhibited by cured blends in different solvent environments can be ascribed to the inclusion of CC-AR in the blend. The amine content, intermolecular attractions of the polymer chains, β-hydroxyurethane hydrogen bonds, and cycloaliphatic content of IPDA can contribute to the enhancement of the interfacial surface of the CJCO/CC-AR blend. The NIPU film with high amine content (30.8% of IPDA) exhibited better solvent and chemical resistance compared with the CJCO/CC-AR blend (ratio of 1:3) and the CJCO film cured with DM.

## Figures and Tables

**Table 1:** Characteristics of jatropha curcas oil compared to other types of vegetable oils.

Oil	C 18:3 (%)	C 18:2 (%)	C 18:1 (%)	RA	SFA	V/mPa s	*M*_w_/g·mol^−1^	AV/mg·KOH·g^−1^
SBO	7–10	51	23	0	14	54.3	879.4	2.7
LSO	56	17	24	0	10.4–11.6	48.4	887.4 [23]	4.0
JCO	0	45.15	39.5	0	14.3	42.3	900.0	2.6
CSO	0.75	3	44	90	1.5	700	927.0 [24]	2.0

SBO = soybean oil, LSO = linseed oil, CSO = castor oil, SFA = saturated fatty acid, *M*_w_ = molecular weight, AV = acid value, RA = ricinoleic acid, V = viscosity.

**Table 2:** The comparison reaction parameters used for cyclic carbonation of various VOs in the presence of TBAB.

Oil	Nature of the Reactor System	*T*/(°C)	*P*/(MPa)	*RT*/(h)	*C*/(%)	Reference
JCO	Pressure reactor	120	2.0	30	99	Present study
ESBO	Flow of CO_2_	110	0.101	70	94	Tamami et al. [27]
ESBO	MI with flow of CO_2_	120	0.101	40	95	Mazo and Rios [29]
ECSO	Autoclave	140	3.000	24	100	Zhang et al. [4]
ESBO	Autoclave/sc CO_2_	100	10.300	20	94	Doll and Erhan [22]
ESBO	Pressure reactor	140	5.650	20	100	Javni et al. [28]
ELO	Pressure reactor	140	3.000	20	100	Bähr and Mülhaupt [43]

*T* = temperature, *P* = CO_2_ pressure, *RT* = reaction time, *C* = conversion, MI = microwave irradiation, ESBO = epoxidized soybean oil, ECBO = epoxidized cottonseed oil, ELO = epoxidized linseed oil.

**Table 3:** The number of linear fatty acid chains of the alkyd resulted in the low amount of carbonate in CJCO and CC-AR.

Carbonate of JCO	^a^ *M* (g·mol^−1^)	^b^ Viscosity 25 °C/mPa·s	Conversion %	^c^ Carbonate Content/wt %
CJCO	1684	2900 ± 430	99	24.9
CC-AR	798	3400 ± 520	97	20.2

^a^ From GPC; ^b^ Measured with Rheometer TA instruments; ^c 1^H-NMR-spectraoscopy.

**Table 4:** Composition and thermo-mechanical properties of the fabricated NIPUs.

Amine	Carbonate	*w*/*w*	CC/wt %	Amine/wt %	MR of A:C	*T*_g_/(°C)	E-modules (MPa)	TS (MPa)	Break (%)
DM	CJCO	1	24.9	17.8	6:1	23	4 ± 2	5 ± 1	230 ± 40
IPDA	CJCO	1	24.9	34.3	9:1	54	150 ± 30	17 ± 2	67 ± 28
DM	CJCO/CC-AR	1:1	22.6	14.7	5:2	22	12 ± 3	11 ± 2	210 ± 20
	CJCO/CC-AR	1:2	21.8	20.5	3:1	25	70 ± 10	8 ± 1	130 ± 10
	CJCO/CC-AR	1:3	21.4	27.3	3:1	21	220 ± 40	10 ± 2	80 ± 30
	CJCO/CC-AR	1:4	21.1	22.3	7:3	27	108 ± 10	5 ± 2	54 ± 30
IPDA	CJCO/CC-AR	1:1	22.6	35.6	4:1	48	27 ± 5	4 ± 0	170 ± 10
	CJCO/CC-AR	1:2	21.8	30.4	3:1	45	390 ± 20	6 ± 0	70 ± 10
	CJCO/CC-AR	1:3	21.4	30.8	4:1	44	680 ± 140	7 ± 2	30 ± 20
	CJCO/CC-AR	1:4	21.1	24.8	7:3	42	259 ± 30	4 ± 2	160 ± 20

DM = 1,3 diaminopropane; IPDA = isophorone diamine; CC = carbonate content; TS = tensile strength; MR = mole ratio; A = amine; C = carbonate.
